# Esports physical exercise/performance matrix 1.0 country factsheets: results and analyses from Serbia

**DOI:** 10.3389/fpubh.2024.1526297

**Published:** 2025-01-23

**Authors:** Xuanyu Zhou, Yuwen Hu, Radenko M. Matic, Stevo Popovic

**Affiliations:** ^1^College of Physical Education, Hunan Normal University, Changsha, China; ^2^Faculty of Sport and Physical Education, University of Novi Sad, Novi Sad, Serbia; ^3^Western Balkan Sport Innovation Lab, Podgorica, Montenegro; ^4^Faculty for Sport and Physical Education, University of Montenegro, Niksic, Montenegro; ^5^Balkan Institute of Science and Innovation, University of Montenegro, Podgorica, Montenegro; ^6^K-CLUB, Korea University, Seoul, Republic of Korea

**Keywords:** esports, physical exercise, performance assessment, health monitoring, Serbia

## Abstract

The Serbian Country Factsheets on Physical Exercise and Performance for Esports Players provide a foundational assessment of nine physical exercise and performance indicators tailored to esports athletes. This study utilizes Serbia’s Esports Physical Exercise/Performance Matrix 1.0 framework, integrating recent peer-reviewed studies and nationally representative secondary data to evaluate these indicators. However, insufficient data was identified, with all indicators scoring zero due to unreliable evidence. This fact highlights a critical need for targeted health initiatives within Serbia’s esports community. Considering the strong correlation between physical fitness and cognitive skills essential for esports success, the findings suggest that prolonged physical inactivity may impair Serbian players’ competitive potential. Serbia risks falling behind in this rapidly evolving field without a coordinated, cross-sectoral approach to health promotion tailored for esports. This factsheet is a pioneering resource for Serbian esports, aiming to stimulate further research, inform policy adjustments, and foster community engagement to build a healthier and more resilient esports population.

## Introduction

The Serbian Country Factsheets on Physical Exercise and Performance for Esports Players represent a pivotal step in addressing Serbia’s esports community’s unique health and performance needs. This report aims to produce a comprehensive, data-driven assessment by evaluating nine key physical exercise and performance indicators specific to esports athletes in Serbia (1). By synthesizing the most pertinent data on physical activity levels, exercise routines, and potential barriers to activity, this factsheet aspires to serve as a foundational resource for shaping future health and performance initiatives.

The passive nature of esports poses significant health risks, with growing evidence linking prolonged inactivity to adverse physical and mental health outcomes. Globally, the majority of esports players fail to meet the World Health Organization’s recommendation of 150–300 min of moderate-intensity physical activity per week. This fact represents a shortfall associated with reduced cognitive function, impaired physical endurance, and an increased likelihood of chronic health issues over time (2–4). Additionally, research has shown that brief, structured physical activity, such as six-minute low-intensity walks during gaming sessions, can improve cognitive planning and peripheral blood flow, mitigating some of the adverse effects of prolonged sitting (5). Despite these findings, many esports players do not prioritize exercise as part of their training regimens (4). Studies indicate that daily sedentary time among esports players ranges from 5.5 to 11 h, comparable to levels observed in high-risk sedentary populations (6–8).

Physical fitness plays a crucial role in esports success, with regular exercise linked to improved cognitive performance and in-game metrics such as reaction time, task accuracy, and decision-making. Nevertheless, research highlights a persistent gap in integrating exercise programs tailored to esports athletes, even among professional players (4, 5). This gap is particularly pronounced in Serbia, primarily due to a need for more research related to this issue but also due to the minimal attention that has been directed toward promoting health and structured physical activity within the esports community.

As the esports industry continues to professionalize, addressing these gaps is imperative. This report provides a detailed snapshot of the current health landscape for Serbian esports players, serving as a pioneering reference for local stakeholders. By advocating for targeted health promotion strategies, cross-sector collaborations, and evidence-based policy adjustments, Serbia’s esports community can foster a healthier, more competitive environment, ensuring resilience in this rapidly evolving global field.

## Methods

This study was conducted by a multidisciplinary team specializing in physical activity assessment and national surveillance. The team, supported by the WBF Move regional project and guided by internationally recognized experts, developed the Serbian Country Factsheets on Physical Exercise and Performance for Esports Players. The research adhered to the Esports Physical Exercise/Performance Matrix 1.0 protocol (1), a systematic framework for evaluating and grading esports players’ physical exercise and performance indicators. This protocol facilitated an objective and standardized data collection and analysis approach, ensuring reliable and reproducible results.

The study focused on nine core physical exercises’ and performance indicators relevant to esports athletes, as specified by the above-mentioned protocol. Primary data sources included peer-reviewed scientific articles, while secondary data sources comprised governmental and nonprofit reports, publicly accessible datasets, and online content. A comprehensive search of electronic databases (SportDiscus, Scopus, PubMed/MEDLINE, and Web of Science) was conducted using precise search syntaxes detailed in the protocol. Additional searches were performed on platforms such as Open Access Theses and Dissertations (OATD), the Networked Digital Library of Theses and Dissertations (NDLTD), and Google to identify supplementary online resources relevant to grading the indicators. Sociodemographic indicators specific to Serbia were also examined to contextualize the findings, utilizing national statistics and reports.

A 10-point grading scale was employed to score each physical exercise and performance indicator, ranging from 10 (exceptional) to 0 (insufficient information). This scoring framework was derived from benchmarks established within the Esports Physical Exercise/Performance Matrix 1.0 protocol. Scores reflected the extent and quality of available data, evaluating whether Serbian esports players met specified physical activity guidelines. Indicators with insufficient data were assigned a zero score, emphasizing areas requiring further research.

Inter-rater reliability was prioritized to ensure consistency in scoring. Coders underwent standardized training on the grading protocol and associated coding standards. Cohen’s kappa coefficient (*κ*) was calculated to assess agreement between coders, yielding an initial value of *κ* = 1, indicating perfect alignment across all indicators.

The analysis began with a comprehensive review of sociodemographic data to construct a demographic profile for Serbia. Subsequently, each physical exercise and performance indicator was evaluated, and scores were assigned based on findings from primary and secondary data sources. Indicators needing more data were assigned a score of zero to reflect the absence of reliable information. Quantitative and qualitative analyses were conducted to contextualize the findings, highlight data gaps, and assess Serbia’s efforts to promote physical activity among esports athletes.

## Results

The Serbian Country Factsheets on Physical Exercise and Performance for Esports Players comprehensively evaluate physical exercise and performance indicators among Serbian esports athletes. The results, organized by indicators, reveal significant gaps in available data, underscoring the urgent need for enhanced research and targeted interventions within this population.

An initial demographic profile of Serbia, presented in Table 1, contextualizes the analysis of esports players’ physical activity. Serbia’s urban population is 57%, with a relatively high internet penetration rate of 84%, suggesting potential access to online resources for health promotion. However, despite these favorable demographics, no nationally representative studies on physical exercise for esports players in Serbia were identified.

**Table 1:** Country’s demographic profile.

Questions	Answers
Country for which you are providing responses:	Serbia
Total population (number of people):	7,149,077
Urban population (%):	57
Human capital index (number between 0 and 1):	0.8
GDP per capita (current US$):	9,537.7
GDP growth (annual %):	2.5
Unemployment, total (% of total labor force):	9.5
Literacy rate, adult total (% of people ages 15 and above):	99
Government expenditure on education, total (% of government expenditure):	7.1
Government expenditure on recreational and sporting services, total (% of government expenditure):	0.4
Individuals using the Internet (% of population):	84
Mobile cellular subscriptions (per 100 people):	124
Fixed broadband subscriptions (per 100 people):	26.19
High-technology exports (current US$):	5
Public health expenditure (% of GDP):	8.73
Life expectancy at birth, total (years):	73
Physical activity prevalence (%):	61
Deaths due to physical inactivity (%):	10
National physical activity plan (Y/N):	Y
Esports national federation (Y/N):	Y

Data were collected from the following website: The World Bank (14) and Global Observatory for Physical Activity (15).

Each of the nine core indicators was graded based on the available evidence, or lack thereof, as shown in Table 2. Due to insufficient data, all indicators received a score of zero. This score indicates a critical lack of documented physical activity practices or initiatives for esports players, indicating a significant gap in health efforts targeting this group. Key findings include (1) Overall Physical Activity/Exercise: No data was available on the percentage of esports players meeting recommended physical activity guidelines. This absence suggests minimal tracking of physical fitness within the esports community; (2) Formal and Informal Physical Activity/Exercise: There was no evidence of organized exercise programs or informal activity habits specific to esports players, which could help mitigate sedentary behavior; (3) Sedentary Behaviors: While general statistics suggest high sedentary rates among esports players globally, there is no data on how Serbian players fare concerning recommended sedentary behavior limits; and (4) Physical Performance and Organization Support: No organizations were found to offer structured physical activity support or certified specialists to guide esports players in Serbia. Furthermore, there is no indication of community or government-led initiatives to improve physical activity among esports players.

**Table 2:** Esports PEP matrix 1.0 indicator and Benchmark grades.

Indicator	Grade	Benchmark
Overall physical Activity/Exercise	0	% of esports players who meet the Canadian Sedentary Behavior Guidelines (16), which recommend that aged group 18–64 years old accumulate at least 150 min of moderate to vigorous aerobic physical activity throughout the week; muscle strengthening activities using major muscle groups at least twice a week; and several hours of light physical activity, including standing.
Formal physical activity/exercise	0	% of esports players who participate in organized (formal) physical activity/exercise programs within esports organization.
Informal physical activity/exercise	0	% of esports players who participate in unorganized (informal) physical activity/exercise at any intensity for more than 2 h a day. % of esports players who report being outdoors for more than 2 h a day, without sitting or lying with low energy expenditure.
Active transportation	0	% of esports players who use active transportation, such as wheeling, walking and cycling, to get to and from places they visit on the daily bases.
Sedentary behaviors	0	% of esports players who meet the Canadian Sedentary Behavior Guidelines (16), which recommend that aged group 18–64 years old spent at most 8 h a day sitting or lying with low energy expenditure, while awake, in the context of occupational, educational, home and community settings, and transportation.
Physical performance	0	Average percentile values achieved on certain physical fitness indicators based on the normative published by Hoffmann et al. (17).
Esports organization	0	% of esports organization with active institutional policies such as daily physical activity/exercise programs. % of esports organizations where the majority of esports players are taught by a certified physical activity/exercise specialists. % of esports organizations where the majority of esports players have regular access to facilities and equipment that support physical activity/exercise.
Community and environment	0	% of esports players who report having physical activity/exercise facilities or programs available to them in their community and environment (e.g., home, neighborhood, school, work, et cetera).
Government	0	% of esports organization that report receiving any funds and resources for the implementation of physical activity/exercise programs for esports players. % of esports organization that report any evidence of leadership in providing any specific type of physical activity/exercise opportunities for esports players. % of esports organization that report any demonstrated progress through the key stages of public policy making.

The inter-judge reliability assessment using Cohen’s kappa coefficient (*κ* = 1) confirmed perfect agreement among coders, indicating a consistent and rigorous grading process. However, the lack of data across indicators highlights a systemic issue in data availability and collection specific to Serbian esports athletes.

The uniform absence of data across all indicators suggests that Serbia’s esports community needs to be more actively engaged in physical activity or health promotion initiatives. This result underscores a need for immediate policy interventions and further research. Identifying targeted strategies to monitor and promote physical fitness within the esports community should be a priority to improve health outcomes and competitive performance.

## Discussion

**Figure 1:**
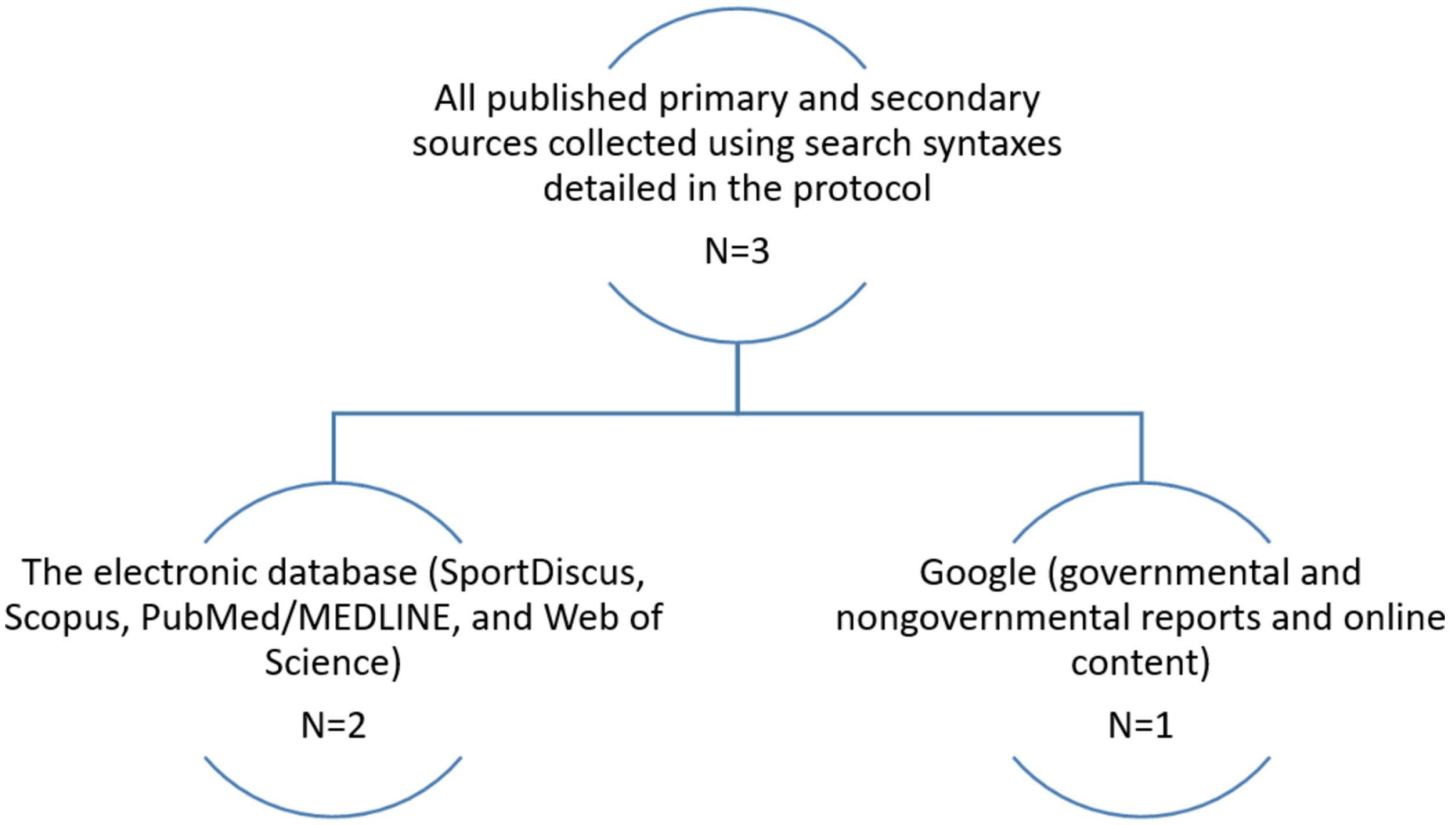
All published sources about all nine indicators for Serbian esports players.

The findings of this report underscore a critical gap in Serbia’s approach to promoting physical activity among esports players, a rapidly growing demographic with distinct health needs worldwide. A review of the available literature identified only three relevant documents on esports-related physical activity and health within Serbia, highlighting the severe lack of attention to this area (Figure 1). These three sources included two articles from electronic databases (9, 10) and one governmental report (11). However, none of these sources contained substantial data to enable the grading of physical exercise and performance indicators specifically for Serbian esports players, and they were excluded from further analyses. This absence is particularly concerning given the broader evidence linking sedentary behavior to adverse health outcomes, which can affect not only players’ physical health but also their cognitive performance, which represents a critical component of esports success (4, 7).

The zero scores across all nine indicators reflect a substantial lack of knowledge about health practices or policies specific to esports in Serbia. The three identified documents provided limited context regarding physical activity and health practices in esports without offering detailed assessments or guidelines tailored to the Serbian esports community. This absence of relevant data underscores a systemic failure to prioritize health monitoring and intervention within esports.

Physical inactivity is a recognized global issue among esports players, with studies reporting high daily sedentary times (6, 8). In countries where esports have been integrated into educational or recreational systems, organized exercise programs and access to trained specialists have helped address these challenges (10). In contrast, Serbian esports players currently lack these foundational supports, leaving them vulnerable to the risks associated with prolonged inactivity.

The results of this study highlight the urgent need for Serbia to initiate as many investigations as possible to help develop strategies that integrate physical activity into esports. National guidelines could be modeled after existing youth-oriented activity recommendations, as Long et al. (1) suggested, to address esports’ unique demands. The Serbian esports community could also benefit from structured physical activity programs implemented within esports organizations, as described by Andjelic et al. (9), who emphasized the potential cognitive benefits of physical exercise for esports players. Additionally, partnerships across sectors such as health, education, and esports organizations could help establish an infrastructure that supports regular physical activity for esports athletes.

Investing in further research is essential to understanding the health needs of esports players in Serbia. Establishing a formal surveillance system to monitor physical activity among Serbian esports players would enable the collection of representative data to inform targeted interventions. Moreover, creating a specialized task force to address esports health could help integrate this community into broader health initiatives, ensuring adequate resources and support (12).

A primary limitation of this study is the need for existing, nationally representative data on Serbian esports players. The three identified articles did not provide sufficient detail to comprehensively assess health outcomes and physical activity behaviors in this community. Future research should prioritize collecting detailed data to fill this gap and develop reliable, culturally relevant health recommendations. Longitudinal studies could further explore the relationship between physical activity levels, cognitive function, and esports performance, as McHugh (13) underscores the importance of reliability in such studies.

Finally, the results underscore the need for Serbia to consider international best practices, potentially adapting frameworks implemented in countries where esports is more established. Comparative analyses could help identify adaptable policies and practices for promoting physical activity and health within Serbian esports. Many authors have indirectly promoted the idea of blending physical and digital experiences in sports, and this represents a relatively new concept referred to as the “phygital” sports concept.

## Conclusion

This brief research report represents an essential first step in documenting and addressing the health needs of Serbian esports players. Without immediate action, this community will remain at risk of health challenges that could undermine their competitive potential and quality of life. By investing in research, policy development, and community health initiatives, Serbia can cultivate a healthier, more resilient esports population and contribute to the sustainable growth of esports as a respected discipline within the broader sports community. Serbia should establish a national surveillance system to monitor physical activity, sedentary behavior, and health outcomes among esports players to address these challenges. Collaborating with universities and research organizations can help ensure the collection of robust, longitudinal data to inform evidence-based interventions. On the other hand, developing and enforcing health guidelines specific to esports players is also critical. These guidelines should incorporate international best practices and emphasize the importance of regular physical activity, proper nutrition, and strategies to mitigate the effects of prolonged sedentary behavior. Esports organizations should be encouraged to adopt these guidelines, with support from fitness specialists who understand the unique demands of esports. Furthermore, structured exercise programs integrated into esports training schedules can help reduce inactivity while improving both physical and cognitive performance, while raising awareness about the importance of health in esports through partnerships with schools, healthcare providers, and esports stakeholders is another vital step. Public health policies should also integrate esports into Serbia’s national physical activity and health strategies. It could involve funding health initiatives, supporting research, and building cross-sector partnerships to ensure sustainable implementation and contribute to the sustainable growth of esports as both a competitive sport and a public health priority.

## Data Availability

The raw data supporting the conclusions of this article will be made available by the authors, without undue reservation.
